# The walking surface influences vertical ground reaction force and centre of pressure data obtained with pressure-sensing insoles

**DOI:** 10.3389/fdgth.2024.1476335

**Published:** 2024-11-08

**Authors:** Elke Warmerdam, Lea-Marie Burger, Diana F. Mergen, Marcel Orth, Tim Pohlemann, Bergita Ganse

**Affiliations:** ^1^ Werner Siemens-Endowed Chair for Innovative Implant Development (Fracture Healing), Clinics and Institutes of Surgery, Saarland University, Homburg, Germany; ^2^ Department of Trauma, Hand and Reconstructive Surgery, Clinics and Institutes of Surgery, Saarland University, Homburg, Germany

**Keywords:** dynamic pedography, ground reaction force, instrumented insoles, mHealth, outdoor, variability

## Abstract

**Background:**

Gait can be continuously monitored via vertical ground reaction force (VGRF) and centre of pressure (COP) measurement with pressure-sensing insoles. During daily living, a variety of walking surfaces will be encountered, which could affect the collected data. These effects might need to be taken into account when analysing disease- or injury-related gait characteristics to prevent misinterpretation, especially when drawing conclusions from data obtained in clinical populations. We hypothesized characteristic changes in insole-derived VGRF and COP parameters of healthy participants when walking on different surfaces.

**Methods:**

Participants walked on flat indoor surface, flat and inclined outdoor surfaces, as well as on forest, gravel, grass, and sand surfaces while wearing pressure-sensing insoles with 16 pressure sensors each at a recording frequency of 100 Hz. Several gait parameters were extracted from the VGRF and COP data, and were compared between surfaces using repeated measures ANOVA.

**Results:**

Thirty participants were included (22 women and 7 men, age 30 ± 12 years, height 172 ± 8 cm, weight 76 ± 23 kg). VGRF and COP data were significantly influenced by the type of surface. The rmANOVA revealed significant within-subject differences between the walking surfaces in all calculated parameters. The largest changes in the VGRF and COP patterns occurred during uphill and downhill walking. Walking on compliant surfaces led to increased gait variability. The highest variability was observed when walking on sand. The change from walking indoors to outdoors, be it on flat, inclined, forest, gravel, grass or sand surfaces, was characterized by a characteristic change in the VGRF stance-phase curve. Based on these characteristic changes, it could be possible to identify whether someone is walking on a slope, as well as on non-compliant or compliant surfaces, while it is difficult to distinguish between different types of compliant surfaces.

**Conclusion:**

VGRF data are affected by the type of walking surface in healthy adults. Walking on a slope affects VGRF and COP parameters, and in addition, the compliance of the surface increases their variability. When analysing gait data measured via insoles during daily living, we recommend to correct for the surface type to decrease variability.

## 1 Introduction

Among the new opportunities currently studied in digital medicine are the benefits of continuous monitoring of changes in gait quality in clinical populations during their daily life (1, 2). These continuous unsupervised assessments provide additional information to clinical assessments where patients tend to show their best performance in a laboratory setting (3). With the daily living data, disease progression and the effect of treatment can be monitored with a greater time resolution and in a real-life setting compared to clinical visits. This information can be used to provide earlier interventions and more individualised treatment. In the development of smart implants that are equipped with sensors to allow continuous gait monitoring in populations such as patients with bone fractures of the tibia, the stance-phase vertical ground reaction force (VGRF) curve is of particular interest to monitor healing (4, 5). To achieve the best results in analysing these long-term data sets, it is important to account for factors that may increase variability and noise. Variations in the VGRF curve among surface types might be among such factors.

The gait in daily life can be monitored with different types of sensors. The instrumented pressure-sensing insoles are among the available wearable sensors and have become available for long-term recordings (6). Most of the available instrumented insoles provide the pressure distribution underneath each foot, the centre of pressure (COP), and the total VGRF. The VGRF curve during the stance phase of walking usually has two maxima with an in-between minimum (7, 8). The VGRF curve is influenced by anthropometric factors, slope of walking surface and muscle strength (9, 10). The VGRF curve shows a different pattern with varying types of locomotion, such as stepping up and down, as well as running (10–13).

Analysing gait has traditionally been conducted indoors on even surfaces or artificially created irregular or inclined surfaces. When conducting continuous measurements in daily life, gait is not only recorded indoors but also outdoors in different contexts. These different contexts, such as varying ground surfaces, can influence the gait pattern. Walking on an irregular surface, which is usually assessed on artificial surfaces in the lab, affected the joint kinematics and increased muscle activity, but especially increased the variability of spatiotemporal parameters, joint kinematics, muscle activity and trunk movements during walking (14–18). Gait variability increased with increasing surface irregularity (18).

When analysing long-term insole data, short-term changes in the gait pattern might be caused by walking on different surface types. Therefore, it might be necessary to correct for the effects of different surfaces to avoid misinterpretation of the data, e.g., an increase in gait variability could be interpreted as a decrease in performance, but it could also be caused by a change in walking surface. The aim of this study was to explore the effect of the surface type on the VGRF and COP data, and to provide an indication about which surface types affect the gait pattern in which ways and need to be taken into account when analysing continuous daily living data. We hypothesized characteristic changes in the VGRF and COP data of healthy participants when walking on flat indoor compared to flat and inclined outdoor surfaces, as well as compared to forest, gravel, grass, and sand surfaces.

## 2 Methods

Ethical approval was obtained from the ethical committee of Saarland Medical Board (Ärztekammer des Saarlandes, Germany, application number 30/21). Informed consent was obtained according to the Declaration of Helsinki. The study is registered in the German Clinical Trials Register (DRKS-ID: DRKS00025108).

### 2.1 Data collection

Healthy adults between 18 and 65 years were included in the study. Adults with gait disorders, use of walking aids, pregnancy or inability to give informed consent were excluded.

**Figure 1:**
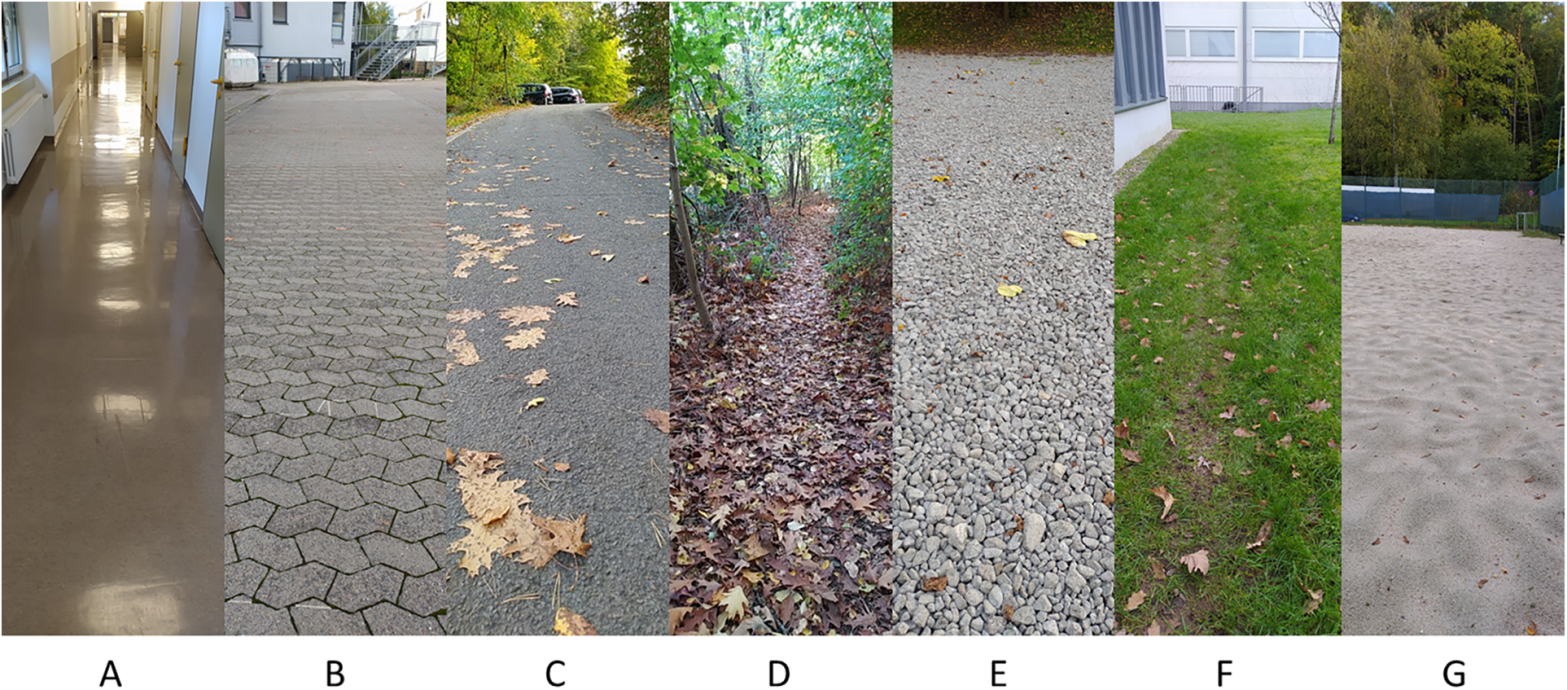
The different types of surfaces that the assessments were performed on. **(A)** Indoor (flat); **(B)** Outdoor flat **(C)**. Uphill and downhill (outdoor); **(D)** Forest; **(E)** Gravel; **(F)** Grass; **(G)** Sand.

Participants were asked to wear trainers, otherwise flat closed shoes without ankle coverage if they had no trainers. The shoes were not standardized, but all had a soft and not a hard shoe sole. Their shoes were fitted with a pair of pressure-sensing insoles (OpenGo, Moticon GmbH, Munich, Germany), that have been proven valid and reliable in previous studies (19). These insoles each contain 16 pressure sensors and an inertial measurement unit (triaxial accelerometer and gyroscope). The insoles were individually calibrated to the participant's body weight. Data were collected with a sample frequency of 100 Hz. The participants walked on eight different surfaces, including one indoors and seven outdoors: flat hallway indoors, flat outdoors, uphill (asphalt; estimated slope 8%), downhill (asphalt; estimated slope −8%), forest (soil ground with tree roots sticking out and fallen leaves and small branches on top), gravel, grass and sand (Figure 1). Apart from the uphill and downhill condition, the other conditions were flat. On each surface the start and the end of a 30 m track were outlined. Participants were asked to start walking at least two steps before the start line and to continue walking for at least two steps at the end of the course. Participants were instructed to walk at their preferred speed. It is known that the walking speed impacts the VGRF data (20). Since the walking speed cannot be accurately measured with instrumented insoles, this effect was not taken into account in this study, but we expect that on average people adapt their speed in a similar fashion to the different conditions. Data collection was started when the participants crossed the start line and stopped when they crossed the 30-m mark. Short breaks between the conditions were present where the participants walked towards the next locations. Data were collected in October and November 2022 at an average daily temperature of ten degrees Celsius and only small amounts of rain to ensure the outdoor surfaces were minimally affected by weather conditions.

### 2.2 Data processing

The pressure sensor data were extracted from the insoles with the OpenGo software (Moticon GmbH, Munich, Germany). The VGRF data and centre of pressure (COP) data were calculated by the software and were then exported for further processing.

**Figure 2:**
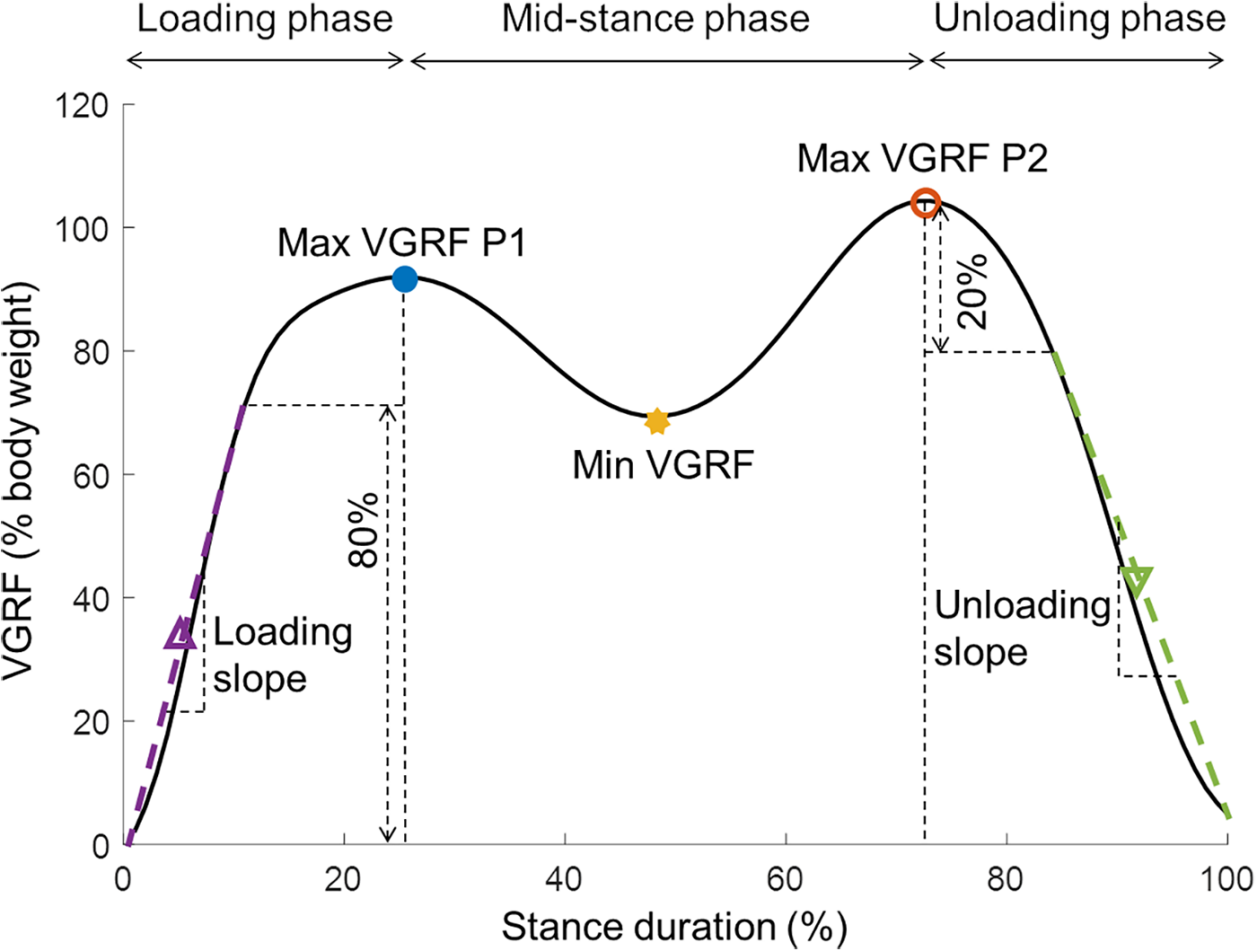
The vertical ground reaction force (VGRF) during the stance phase with the extracted parameters. Max, maximal; Min, minimal; P, peak; VGRF, vertical ground reaction force.

The pressure sensor data were extracted from the insoles with the OpenGo software (Moticon GmbH, Munich, Germany). The VGRF data and centre of pressure (COP) data were calculated by the software and were exported for further processing. A custom-made MATLAB script was used to process the data and calculate the gait parameters. Data gaps of up to 17 milliseconds were present in a few measurements and were filled using spline interpolation. Data were filtered with a fourth-order Butterworth filter with a cut-off frequency of 10 Hz. Next, stance phases were detected. A stance phase was defined as a consecutive VGRF reading above 30 N with at least 0.45 s between one end of the swing phase and the end of the next swing phase of the same leg. Each stance phase was time-normalised using linear interpolation and the VGRF data were normalised to percentage bodyweight. Two local maxima with an inter-peak distance of 30% of the normalised stance phase time were detected during each stance phase. The minimum VGRF between these two local maxima was also extracted. The loading slope was calculated from the start of the stance phase up to 80% of the normalised VGRF of the first maximum (11). The unloading slope was calculated from the moment that the VGRF of the second maximum was decreased by 20% up to the end of the stance phase (Figure 2). The maximal forward-travelled distance of the COP underneath the foot during the loading phase was calculated and will be referred to as the COP length. From all the above-mentioned parameters, the coefficient of variation was calculated as a measure of variability. All parameters were calculated for both the left and right foot and averaged for each foot, and the average of these two values was used in the data analysis.

### 2.3 Statistics

Statistical tests were performed with JASP (version 0.16.4; https://jasp-stats.org/). The parameters were tested for normality with the Shapiro-Wilk test. The loading slope data were not normally distributed and therefore transformed ($y=\;x^{-1}$). A one-way repeated measures ANOVA was conducted with the different surface types as a within-subjects factor. In case sphericity was violated, the Greenhouse-Geisser correction was used. Eta squared was calculated as a measure of effect size. Post-hoc testing was performed according to the Holm method. Significance was defined as *p* < 0.05.

## 3 Results

Thirty healthy adults were included in the study (Table 1). Data of one participant were discarded because of zeroing issues in the proprietary insole algorithm during the swing phases.

**Table 1:** Demographics of the participants (mean ± standard deviation).

*N* (female)	29 (22)
Age (years)	30 ± 12
Height (cm)	172 ± 8
Weight (kg)	76 ± 23

**Figure 3:**
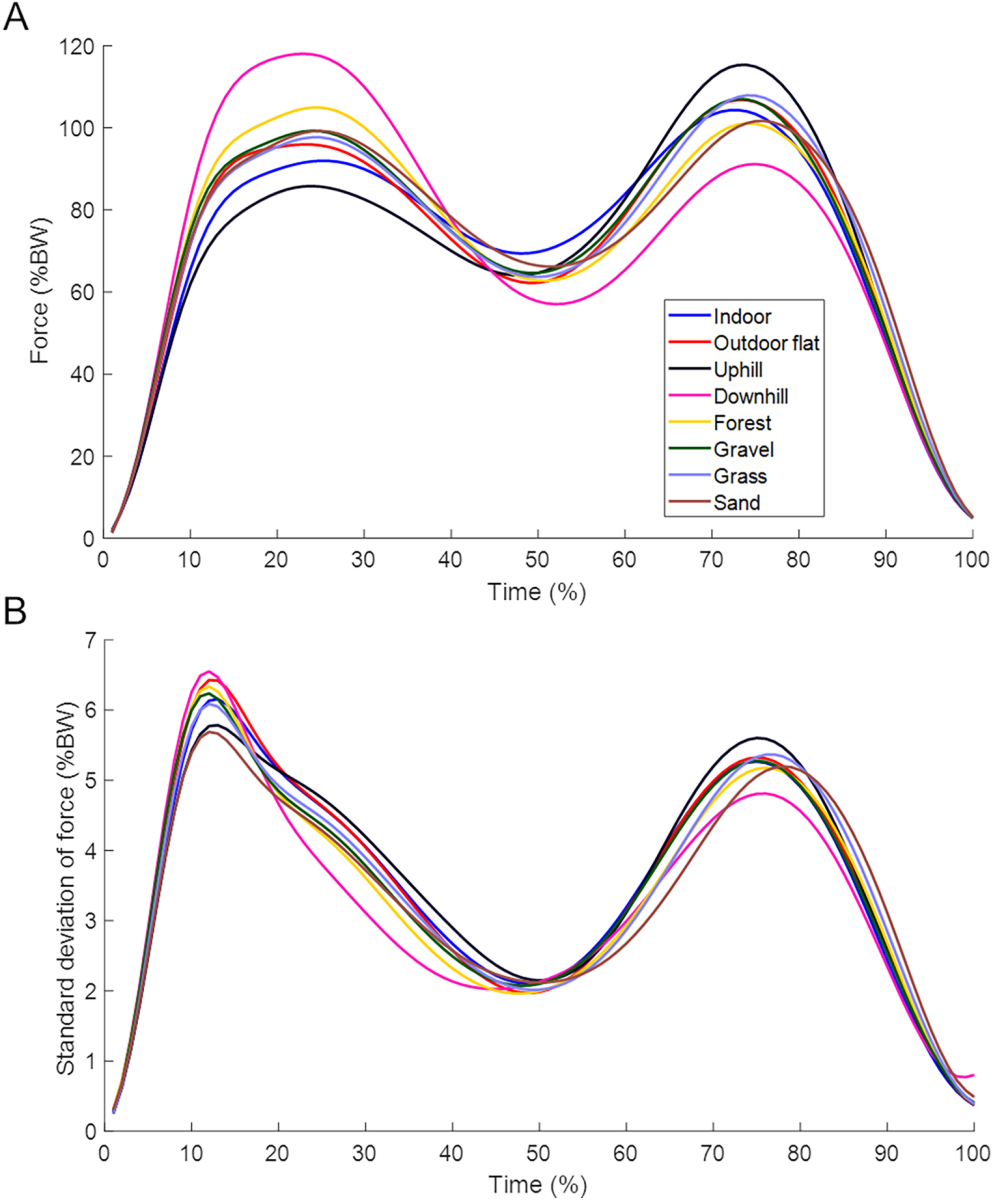
**(A)** Time and body weight-normalised vertical ground reaction curve during the stance phase per surface type. **(B)** Standard deviations of the normalised vertical ground reaction curves during the stance phase per surface type. Stance phase trajectories were averaged across participants.

**Figure 4:**
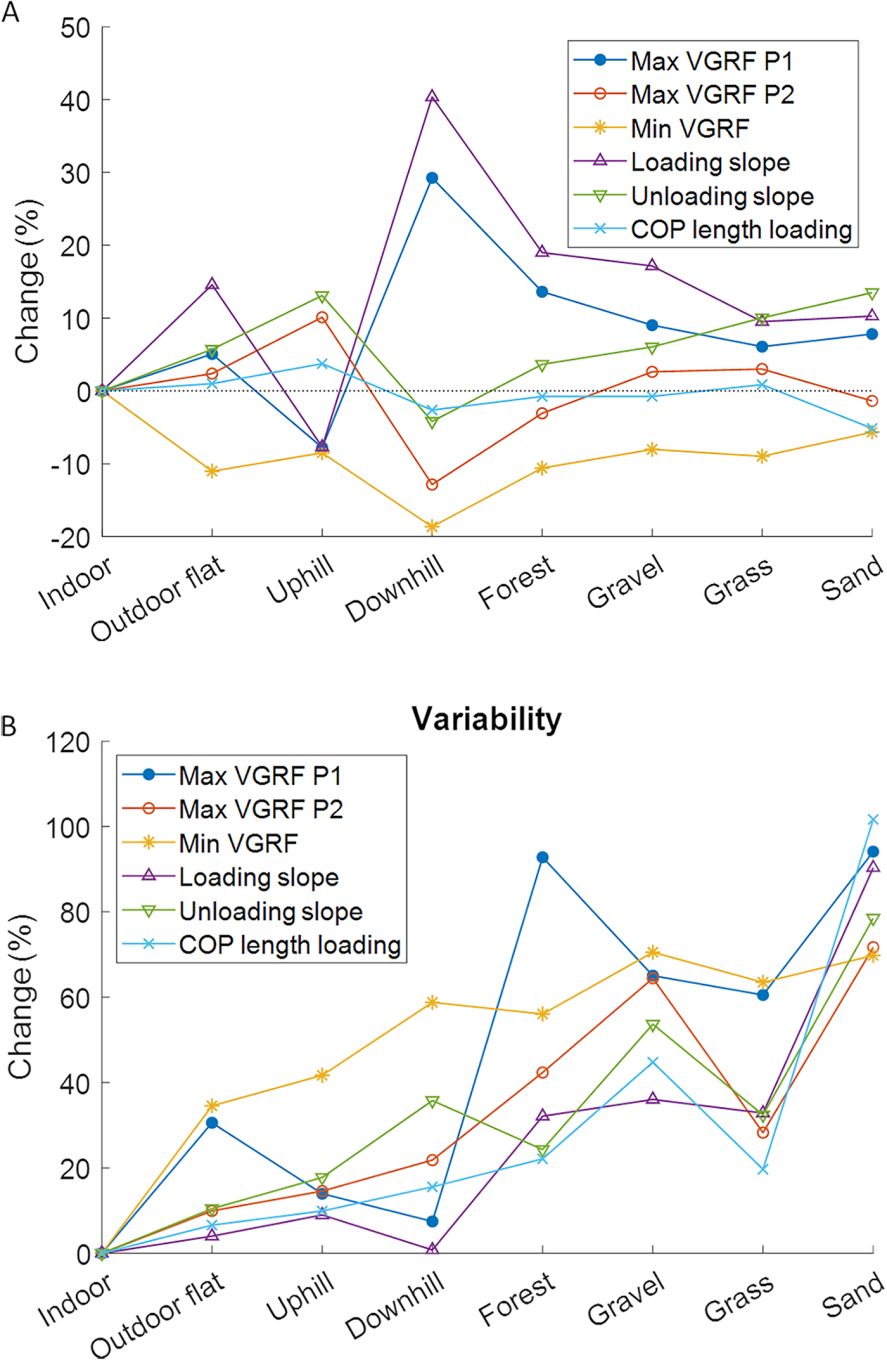
Percent changes relative to indoor walking for the averaged gait parameters **(A)** and the variability of the gait parameters **(B)**. COP, center of pressure; max, maximal; min, minimal; P, peak; VGRF, vertical ground reaction.

The VGRF curves for the different surfaces are shown in Figure 3 and the obtained parameters are presented in Table 2. Figure 4 indicates relative changes in parameters compared to indoor walking.

**Table 2:** Mean and standard deviation of the obtained parameters per surface type.

	Indoor	Outdoor flat	Uphill	Downhill	Forest	Gravel	Grass	Sand	*p*-value RMA (effect size)
Max VGRF P1 (%BW)	95.4 ± 7.7	100.3 ± 10.3	88.7 ± 8.6	123.2 ± 12.5	108.9 ± 9.9	103.6 ± 10.8	101.4 ± 9.3	103.5 ± 10.4	<.001 (0.88)
Max VGRF P2 (%BW)	107.2 ± 10.0	110.0 ± 12.2	118.4 ± 13.9	93.8 ± 9.6	103.8 ± 10.1	110.2 ± 12.4	110.9 ± 12.5	105.6 ± 11.7	<.001 (0.80)
Min VGRF (%BW)	67.0 ± 6.7	59.7 ± 8.0	61.3 ± 7.7	55.0 ± 8.3	60.4 ± 7.6	61.8 ± 6.9	61.2 ± 7.3	63.8 ± 5.7	<.001 (0.54)
Loading slope (%BW/%T)	5.0 ± 1.4	5.7 ± 1.7	4.6 ± 1.1	7.0 ± 2.0	5.9 ± 1.7	5.4 ± 1.4	5.4 ± 1.4	5.5 ± 1.4	<.001 (0.67)
Unloading slope (%BW/%T)	−4.4 ± 0.6	−4.6 ± 0.7	−5.0 ± 0.8	−4.2 ± 0.6	−4.5 ± 0.7	−4.7 ± 0.7	−4.9 ± 0.7	−5.0 ± 0.9	<.001 (0.54)
COP length loading phase (AU)	0.304 ± 0.017	0.307 ± 0.019	0.314 ± 0.017	0.298 ± 0.025	0.302 ± 0.025	0.303 ± 0.023	0.307 ± 0.020	0.288 ± 0.021	<.001 (0.38)
Variability max VGRF P1 (%BW)	0.040 ± 0.014	0.052 ± 0.016	0.045 ± 0.012	0.043 ± 0.010	0.078 ± 0.015	0.067 ± 0.013	0.064 ± 0.013	0.078 ± 0.016	<.001 (0.60)
Variability max VGRF P2 (%BW)	0.035 ± 0.008	0.038 ± 0.008	0.041 ± 0.014	0.043 ± 0.013	0.050 ± 0.016	0.057 ± 0.012	0.045 ± 0.011	0.061 ± 0.016	<.001 (0.44)
Variability min VGRF (%BW)	0.045 ± 0.010	0.061 ± 0.019	0.065 ± 0.021	0.073 ± 0.023	0.071 ± 0.027	0.077 ± 0.026	0.073 ± 0.021	0.075 ± 0.025	<.001 (0.27)
Variability loading slope (%BW/%T)	0.114 ± 0.050	0.121 ± 0.048	0.124 ± 0.046	0.116 ± 0.040	0.149 ± 0.044	0.156 ± 0.052	0.150 ± 0.048	0.216 ± 0.070	<.001 (0.45)
Variability unloading slope (%BW/%T)	−0.063 ± 0.014	−0.070 ± 0.029	−0.072 ± 0.031	−0.086 ± 0.029	−0.077 ± 0.019	−0.095 ± 0.024	−0.080 ± 0.024	−0.107 ± 0.030	<.001 (0.36)
Variability COP length loading phase (AU)	0.064 ± 0.019	0.070 ± 0.017	0.070 ± 0.013	0.074 ± 0.012	0.078 ± 0.022	0.092 ± 0.021	0.076 ± 0.013	0.103 ± 0.026	<.001 (0.34)

AU, arbitrary units; COP, center of pressure; max, maximal; min, minimal; P, peak; RMA, repeated measures ANOVA; T, time; VGRF, vertical ground reaction force.

### 3.1 Average values

The repeated measures ANOVA showed significant within-subject differences between the walking surfaces in all calculated parameters (Table 2 and Additional File 1). The largest effects were found for the peaks in the VGRF. *Post hoc* tests showed that for the first peak in the VGRF, the indoor, uphill, downhill and forest surfaces were significantly different from all other surfaces (Additional File 1 Table S1). Downhill walking had the highest first peak in the VGRF and uphill walking the lowest (Table 2 and Figure 4A). For the second peak in the VGRF, uphill walking was significantly higher and downhill walking was significantly lower than all other surfaces (Table 2 and Additional File 1 Table S2). For the minimal VGRF, indoor walking was significantly higher and downhill walking significantly lower than all other surfaces (Table 2 and Additional File 1 Table S3). For the loading slope, it was indoor, uphill and downhill walking that were significantly different from all other surfaces (Additional File 1 Table S4). The lowest loading slope was found for uphill walking followed by indoor walking, and the highest value was found for downhill walking (Table 2). For the unloading slope, most significant differences with other surfaces were found for downhill walking, which was significantly lower than all other surfaces except for indoor walking (Table 2 and Additional File 1 Table S5). For the COP length during the loading phase, sand was significantly lower compared to all other surfaces (Table 2 and Additional File 1 Table S6). The effect sizes were largest for downhill walking in all parameters, except for the COP length during the loading phase, in which case sand had the largest effect sizes (Additional File 1).

### 3.2 Variability values

Figure 4B illustrates changes in variability among surfaces. The highest variability in all parameters, except for the local minimum in the VGRF, was observed when walking on sand (Table 2). The variability of the minimal VGRF during indoor walking and the loading slope during walking on sand were significantly higher compared to all other surfaces (Additional File 1 Table S9 and S10). The largest effect sizes were found for walking on sand for all parameters except the local minimum in the VGRF (Additional File 1).

### 3.3 Percentage changes

The percentage change relative to indoor walking was calculated for each parameter to show how much the parameters changed compared to the most basic flat indoor condition (Figure 4A). The change from walking indoors to outdoors, be it to flat, forest, gravel, grass or sand surfaces, was characterized by a simultaneous increase in the first maximum of the VGRF, loading slope and unloading slope, and a simultaneous decrease in local minimum of the VGRF. While the pattern of parameter changes was clearly distinguished for uphill and downhill walking, forest, gravel, grass and sand showed very similar pattern changes. The variability increased in all conditions compared to indoor walking. The largest increases were found for the compliant surfaces, forest, gravel, grass and sand.

## 4 Discussion

In this study, effects of different walking surfaces on the VGRF and the COP-curve of the gait cycle measured by instrumented insoles were explored. Significant differences in extracted parameters were found for walking on different surfaces with the largest effects on the peaks in the VGRF. Differences between conditions were especially seen in level surfaces and surfaces with a slope for the average gait parameters. The largest effect sizes (Additional File 1) were found for downhill walking, except for COP length where the largest effect size was found for uphill walking. The gait variability was mainly affected by the compliance of the surface, where the largest effects were found on sand.

When analysing continuous insole data obtained during daily living, characteristic changes in combined parameter patterns may indicate uphill and downhill walking, as well as walking on a more or less compliant surface. As expected, based on previous studies, uphill and downhill walking lead to characteristic differences in gait patterns compared to level walking (21, 22). These differences were more pronounced during downhill walking than uphill walking, which was comparable with earlier findings (22, 23). Since walking on a slope had a substantial effect on the gait pattern, we recommend that it should be considered when analysing daily living gait data. This is especially relevant for real-time analyses performed on devices that deliver alarms as treatment intervention, such as applying too much load. Estimating the slope that participants are walking on during daily life can be done with the data of an IMU, e.g., by using the accelerometer as a tilt sensor (24). Most commercially available pressure sensing insoles also contain an IMU, making it a feasible option.

Automatic classification of walking surface is required when the surface needs to be taken into account when interpreting the data. Machine learning has already been used to classify different surfaces during walking, but using IMU data (25–27). Instrumented insole data has so far only been used to distinguish between activities with varying success (21, 28). We expect that it is possible to automatically distinguish between different types of surfaces based on VGRF data. Parameters that seem especially suited for this are the ratio between the two maxima in the VGRF to distinguish uphill, downhill and flat surfaces from each other. Variability parameters, which substantially increased when walking on compliant surfaces and increased with compliance of the surface, because postural control is more challenged, might be key in distinguishing between compliant and non-compliant surfaces. Demographic variables should also be added to the machine learning model, because these have an effect on the VGRF (9).

When analysing variability of the gait pattern from daily life measurements, the increased variability when walking on compliant surfaces could lead to mistakes in data interpretation. For example, in patients with Parkinson's disease an increase in gait variability could indicate walking on compliant surfaces or could be an indication that a new dose of dopaminergic medication is required if they are walking on noncompliant surfaces (29). In addition, the correction for the type of surface might depend on and be of interest for further population-related parameters. It is likely that frail people avoid the extra challenges of walking on compliant surfaces. The avoidance of compliant surfaces might be an additional parameter as well as an indication of psychological aspects, such as the fear of falling (30).

Previous studies showed that the effect of irregular surface on gait was larger in older adults compared to younger adults (15, 17). Our study was performed with a young population; therefore, it might be worthwhile to study the effect of walking on irregular surfaces in an older population. This could also be the case for clinical populations with injuries and movement disorders. In clinical populations, short-term changes in the gait pattern could just as well be caused by, e.g., medical conditions, effects of treatments and interventions. Especially, in these clinical populations, it is important to distinguish whether the changes in the gait pattern were caused by something related to the medical condition or by a change in surface to be able to correctly interpret the data.

This study has some limitations. The measurements were mainly performed outside, making them more ecologically valid, however only straight walking was measured. During daily life, people will perform curved walking, as well as additional movements or tasks which could influence the parameters differently. Moreover, we did not take gait speed into account. It could be that the gait speed differed per surface type, which might have influenced the VGRF. However, a previous study did not find differences in gait speed between different surface types (18), therefore we expect that this had only a minor influence on our results. The study had a sex imbalance, because most participants were female. As the VGRF data were normalized to body weight, we expect that the sex imbalance only had a limited effect on the results.

## 5 Conclusions

The gait pattern as assessed by pressure-sensing insoles is influenced by the type of walking surface. Walking on a slope affects VGRF and COP-derived gait parameters, and in addition, the compliance of the surface increases the variability of these parameters. The change from walking indoors to outdoors, be it on flat, inclined, forest, gravel, grass or sand surfaces, is characterized by a typical increase or decrease in multiple gait parameters. When analysing gait data measured via insoles during daily living, we recommend to distinguish between flat and inclined surfaces, as well as non-compliant and compliant surfaces.

## Data Availability

The raw data supporting the conclusions of this article will be made available by the authors, without undue reservation.
