# Supplementary material for: The walking surface influences vertical ground reaction force and centre of pressure data obtained with pressure-sensing insoles
Source: Front Digit Health. 2024 Nov 8;6:1476335. doi: 10.3389/fdgth.2024.1476335 (PMC11582027; doi:10.3389/fdgth.2024.1476335)
Supplement: Supplementary file 1 [file Datasheet1.pdf]

Additional file 1

For each parameter, the result of the one-way repeated measures ANOVA with surface type as within subject factor is provided. The p-values of the post hoc tests (Holm method) are provided in the left lower part of each table (bold if significant) and the effect sizes (Cohen's d) are provided in the right upper part of each table (darker colour represents a higher effect size; 0.2 = small; .5 = medium; .8 = large; 1.2 = very large effect size).

Average values

The first peak in the maximal vertical ground reaction force was significantly different between the surface types ($F_{3.62,\ 101.47}$ = 207.35, p = <.001).

Table 1. The p-values and effect sizes of the post hoc comparisons for the first peak in the maximal vertical ground reaction force.

	Indoor flat	Outdoor flat	Uphill	Downhill	Forest	Gravel	Grass	Sand
Indoor flat		-0.483	0.667	-2.772	-1.344	-0.822	-0.594	-0.811
Outdoor flat	**<.001**		1.150	-2.289	-0.861	-0.339	-0.110	-0.328
Uphill	**<.001**	**<.001**		-3.439	-2.010	-1.488	-1.260	-1.477
Downhill	**<.001**	**<.001**	**<.001**		1.428	1.951	2.179	1.962
Forest	**<.001**	**<.001**	**<.001**	**<.001**		0.522	0.750	0.533
Gravel	**<.001**	**.005**	**<.001**	**<.001**	**<.001**		0.228	0.011
Grass	**<.001**	.532	**<.001**	**<.001**	**<.001**	.089		-0.217
Sand	**<.001**	**.006**	**<.001**	**<.001**	**<.001**	.912	.089	

The second peak in the maximal vertical ground reaction force was significantly different between the surface types ($F_{3.84,\ 107.40}$ = 108.78, p = <.001).

Table 2. The p-values and effect sizes of the post hoc comparisons for the second peak in the maximal vertical ground reaction force.

	Indoor flat	Outdoor flat	Uphill	Downhill	Forest	Gravel	Grass	Sand
Indoor flat		-0.236	-0.968	1.147	0.287	-0.265	-0.318	0.138
Outdoor flat	**0.028**		-0.732	1.383	0.523	-0.029	-0.082	0.374
Uphill	**<.001**	**<.001**		2.115	1.255	0.703	0.650	1.106
Downhill	**<.001**	**<.001**	**<.001**		-0.860	-1.412	-1.465	-1.009
Forest	**0.005**	**<.001**	**<.001**	**<.001**		-0.552	-0.605	-0.149
Gravel	**0.011**	1.000	**<.001**	**<.001**	**<.001**		-0.053	0.403
Grass	**0.001**	0.962	**<.001**	**<.001**	**<.001**	1.000		0.455
Sand	0.382	**<.001**	**<.001**	**<.001**	0.356	**<.001**	**<.001**	

The local minimum in the vertical ground reaction force was significantly different between the surface types ($F_{4.47, 125.06}$ = 33.39, p = <.001).

Table 3. The p-values and effect sizes of the post hoc comparisons for the local minimum in the vertical ground reaction force.

	Indoor flat	Outdoor flat	Uphill	Downhill	Forest	Gravel	Grass	Sand
Indoor flat		1.008	0.784	1.649	0.911	0.723	0.796	0.443
Outdoor flat	**<.001**		-0.224	0.641	-0.098	-0.285	-0.212	-0.566
Uphill	**<.001**	0.479		0.864	0.126	-0.061	0.011	-0.342
Downhill	**<.001**	**<.001**	**<.001**		-0.738	-0.926	-0.853	-1.206
Forest	**<.001**	1.000	1.000	**<.001**		-0.187	-0.115	-0.468
Gravel	**<.001**	.156	1.000	**<.001**	.739		0.073	-0.281
Grass	**<.001**	.533	1.000	**<.001**	1.000	1.000		-0.353
Sand	**.002**	**<.001**	**.040**	**<.001**	**<.001**	.156	**.032**	

The loading slope was significantly different between the surface types ($F_{3.87, 108.28}$ = 56.52, p = <.001).

Table 4. The p-values and effect sizes of the post hoc comparisons for the loading slope.

	Indoor flat	Outdoor flat	Uphill	Downhill	Forest	Gravel	Grass	Sand
Indoor flat		0.562	-0.304	1.362	0.739	0.621	0.444	0.508
Outdoor flat	**<.001**		-0.865	0.800	0.177	0.060	-0.118	-0.054
Uphill	**.014**	**<.001**		1.665	1.043	0.925	0.747	0.812
Downhill	**<.001**	**<.001**	**<.001**		-0.623	-0.741	-0.918	-0.854
Forest	**<.001**	.465	**<.001**	**<.001**		-0.118	-0.295	-0.231
Gravel	**<.001**	1.000	**<.001**	**<.001**	1.000		-0.178	-0.113
Grass	**<.001**	1.000	**<.001**	**<.001**	**.018**	.465		0.064
Sand	**<.001**	1.000	**<.001**	**<.001**	.125	1.000	1.000	

The unloading slope was significantly different between the surface types ($F_{3.46, 96.89}$ = 32.91, p = <.001).

Table 5. The p-values and effect sizes of the post hoc comparisons for the unloading slope.

	Indoor flat	Outdoor flat	Uphill	Downhill	Forest	Gravel	Grass	Sand
Indoor flat		0.343	0.832	-0.241	0.197	0.371	0.641	0.791
Outdoor flat	**.003**		0.489	-0.584	-0.145	0.028	0.298	0.448
Uphill	**<.001**	**<.001**		-1.073	-0.635	-0.461	-0.191	-0.041
Downhill	.084	**<.001**	**<.001**		0.439	0.612	0.882	1.032
Forest	.252	.438	**<.001**	**<.001**		0.174	0.444	0.594
Gravel	**.001**	1.000	**<.001**	**<.001**	.324		0.270	0.420
Grass	**<.001**	**.016**	.253	**<.001**	**<.001**	**.038**		0.150
Sand	**<.001**	**<.001**	1.000	**<.001**	**<.001**	**<.001**	.438	

The center of pressure length during the loading phase was significantly different between the surface types ($F_{4.19, 117.24}$ = 17.20, p = <.001).

Table 6. The p-values and effect sizes of the post hoc comparisons for the center of pressure length during the loading phase.

	Indoor flat	Outdoor flat	Uphill	Downhill	Forest	Gravel	Grass	Sand
Indoor flat		-0.134	-0.467	0.311	0.120	0.081	-0.104	0.790
Outdoor flat	1.000		-0.333	0.445	0.254	0.215	0.030	0.924
Uphill	**.005**	.119		0.778	0.587	0.548	0.362	1.257
Downhill	.180	**.008**	**<.001**		-0.191	-0.230	-0.415	0.479
Forest	1.000	.523	**<.001**	1.000		-0.039	-0.225	0.670
Gravel	1.000	.785	**<.001**	.747	1.000		-0.186	0.709
Grass	1.000	1.000	.063	**.017**	.747	1.000		0.894
Sand	**<.001**	**<.001**	**<.001**	**.003**	**<.001**	**<.001**	**<.001**	

Variability values

The variability of the first peak in the maximal vertical ground reaction force was significantly different between the surface types ($F_{3.92, 109.70}$ = 42.31, p = <.001).

Table 7. The p-values and effect sizes of the post hoc comparisons for the variability of the first peak in the maximal vertical ground reaction force.

	Indoor flat	Outdoor flat	Uphill	Downhill	Forest	Gravel	Grass	Sand
Indoor flat		-0.914	-0.394	-0.263	-2.771	-1.956	-1.778	-2.802
Outdoor flat	**0.003**		0.521	0.652	-1.857	-1.042	-0.864	-1.888
Uphill	0.538	0.203		0.131	-2.378	-1.563	-1.385	-2.408
Downhill	1.000	0.057	1.000		-2.509	-1.694	-1.516	-2.539
Forest	**<.001**	**<.001**	**<.001**	**<.001**		0.815	0.993	-0.031
Gravel	**<.001**	**<.001**	**<.001**	**<.001**	0.008		0.178	-0.846
Grass	**<.001**	**0.005**	**<.001**	**<.001**	**<.001**	1.000		-1.024
Sand	**<.001**	**<.001**	**<.001**	**<.001**	1.000	**0.006**	**<.001**	

The variability of the second peak in the maximal vertical ground reaction force was significantly different between the surface types ($F_{4.77, 133.62}$ = 22.34, p = <.001).

Table 8. The p-values and effect sizes of the post hoc comparisons for the variability of the second peak in the maximal vertical ground reaction force.

	Indoor flat	Outdoor flat	Uphill	Downhill	Forest	Gravel	Grass	Sand
Indoor flat		-0.232	-0.414	-0.620	-1.171	-1.693	-0.784	-2.004
Outdoor flat	1.000		-0.182	-0.388	-0.938	-1.461	-0.552	-1.772
Uphill	0.466	1.000		-0.206	-0.757	-1.279	-0.370	-1.591
Downhill	**0.049**	0.544	1.000		-0.550	-1.073	-0.164	-1.384
Forest	**<.001**	**<.001**	**0.006**	0.117		-0.523	0.387	-0.834
Gravel	**<.001**	**<.001**	**<.001**	**<.001**	0.143		0.909	-0.311
Grass	**0.004**	0.117	0.544	1.000	0.544	**<.001**		-1.221
Sand	**<.001**	**<.001**	**<.001**	**<.001**	**0.002**	0.714	**<.001**	

The variability of the local minimum in the vertical ground reaction force was significantly different between the surface types ($F_{7, 196} = 10.294$, p = <.001).

Table 9. The p-values and effect sizes of the post hoc comparisons for the variability of the local minimum in the vertical ground reaction force.

	Indoor flat	Outdoor flat	Uphill	Downhill	Forest	Gravel	Grass	Sand
Indoor flat		-0.730	-0.896	-1.282	-1.191	-1.451	-1.277	-1.366
Outdoor flat	**0.015**		-0.167	-0.553	-0.462	-0.722	-0.547	-0.636
Uphill	**<.001**	1.000		-0.386	-0.295	-0.555	-0.381	-0.469
Downhill	**<.001**	0.177	0.970		0.091	-0.169	0.006	-0.083
Forest	**<.001**	0.453	1.000	1.000		-0.260	-0.086	-0.175
Gravel	**<.001**	**0.016**	0.177	1.000	1.000		0.174	0.085
Grass	**<.001**	0.177	0.970	1.000	1.000	1.000		-0.089
Sand	**<.001**	0.059	0.440	1.000	1.000	1.000	1.000	

The variability of the loading slope was significantly different between the surface types ($F_{5.04, 141.05} = 22.77$, p = <.001).

Table 10. The p-values and effect sizes of the post hoc comparisons for the variability of the loading slope.

	Indoor flat	Outdoor flat	Uphill	Downhill	Forest	Gravel	Grass	Sand
Indoor flat		-0.124	-0.197	-0.022	-0.674	-0.817	-0.696	-2.014
Outdoor flat	1.000		-0.073	0.102	-0.550	-0.692	-0.571	-1.890
Uphill	1.000	1.000		0.174	-0.477	-0.620	-0.499	-1.817
Downhill	1.000	1.000	1.000		-0.652	-0.794	-0.673	-1.992
Forest	**0.014**	0.073	0.170	**0.018**		-0.142	-0.022	-1.340
Gravel	**0.001**	**0.011**	**0.029**	**0.002**	1.000		0.121	-1.197
Grass	**0.011**	0.057	0.140	**0.014**	1.000	1.000		-1.318
Sand	**<.001**	**<.001**	**<.001**	**<.001**	**<.001**	**<.001**	**<.001**	

The variability of the unloading slope was significantly different between the surface types ($F_{4.71, 131.84} = 15.82$, p = <.001).

Table 11. The p-values and effect sizes of the post hoc comparisons for the variability of the unloading slope.

	Indoor flat	Outdoor flat	Uphill	Downhill	Forest	Gravel	Grass	Sand
Indoor flat		0.289	0.361	0.892	0.556	1.238	0.680	1.723
Outdoor flat	0.874		0.072	0.603	0.267	0.950	0.391	1.434
Uphill	0.696	1.000		0.531	0.195	0.878	0.319	1.362
Downhill	**<.001**	**0.042**	0.103		-0.336	0.347	-0.212	0.831
Forest	0.078	0.894	1.000	0.730		0.683	0.124	1.167
Gravel	**<.001**	**<.001**	**<.001**	0.730	**0.012**		-0.559	0.485
Grass	**0.012**	0.546	0.761	1.000	1.000	0.078		1.043
Sand	**<.001**	**<.001**	**<.001**	**<.001**	**<.001**	0.182	**<.001**	

The variability of the center of pressure length during the loading phase was significantly different between the surface types ($F_{4.90, 137.32} = 14.22$, p = <.001).

Table 12. The p-values and effect sizes of the post hoc comparisons for the variability of the center of pressure length during the loading phase.

	Indoor flat	Outdoor flat	Uphill	Downhill	Forest	Gravel	Grass	Sand
Indoor flat		-0.296	-0.313	-0.529	-0.739	-1.522	-0.655	-2.087
Outdoor flat	1.000		-0.017	-0.233	-0.444	-1.226	-0.359	-1.791
Uphill	1.000	1.000		-0.216	-0.427	-1.209	-0.342	-1.774
Downhill	0.578	1.000	1.000		-0.211	-0.993	-0.126	-1.558
Forest	0.083	1.000	1.000	1.000		-0.782	0.084	-1.348
Gravel	**<.001**	**<.001**	**<.001**	**0.004**	0.053		0.867	-0.565
Grass	0.195	1.000	1.000	1.000	1.000	**0.020**		-1.432
Sand	**<.001**	**<.001**	**<.001**	**<.001**	**<.001**	0.445	**<.001**	
